# A Personalized Approach to Parkinson’s Disease Patients Based on Founder Mutation Analysis

**DOI:** 10.3389/fneur.2016.00071

**Published:** 2016-05-10

**Authors:** Nir Giladi, Anat Mirelman, Avner Thaler, Avi Orr-Urtreger

**Affiliations:** ^1^Laboratory for Early Markers of Neurodegeneration, Department of Neurology, Tel-Aviv Sourasky Medical Center, Tel Aviv University, Tel Aviv, Israel; ^2^Sackler School of Medicine, Tel Aviv University, Tel Aviv, Israel; ^3^Genetic Institute, Tel Aviv Sourasky Medical Center, Tel Aviv, Israel

**Keywords:** Parkinson’s disease, *GBA*, *LRRK2*, phenotype, personalized medicine

## Abstract

While the phenotype of Parkinson disease (PD) is heterogeneous, treatment approaches are mostly uniform. Personalized medicine aims to treat diseases with targeted therapies based on cumulative variables, including genotype. We believe that sufficient evidence has accumulated to warrant the initiation of personalized medicine in PD based on subjects genotype and provide examples for our reasoning from observations of *GBA* and *LRRK2* mutations carriers. While PD patients who carry the G2019S mutation in the *LRRK2* gene seem to develop relatively mild disease with more frequent postural instability gait disturbance phenotype, carriers of mutations in the *GBA* gene tend to have an early onset, rapidly deteriorating disease, with more pronounced cognitive and autonomic impairments. These characteristics have significant implications for treatment and outcome and should be addressed from an early stage in the attempt to improve the patient’s quality of life.

## Introduction

Parkinson disease (PD) is a complex neurodegenerative disorder affecting older adults. The etiology of PD is heterogeneous, genetic, and multi-factorial, resulting in a highly variable clinical course, spanning from a slowly progressive, benign course to a rapidly progressive, disabling disease (1, 2).

Parkinson disease is a highly variable disease; it can start early in the 3rd or 4th decade of life, or as late as the 10th decade, the predominant symptom can be tremor, or the disease phenotype might be non-tremulous with gait, postural impairments, or rigidity as the main features. PD can involve late or early cognitive and behavioral changes, early or late autonomic disturbances, and various manifestation of pain or sleep disturbances (3–5). However, in spite of this large diversity in clinical features, the therapeutic approach, as reflected in therapeutic guidelines, is uniform and symptom oriented, with the exception of age and cognitive state as factors influencing clinical decisions (6, 7). Recent understanding of the significant contribution of multiple gene loci to the risk of developing PD, as well as to the disease phenotypic clinical course, raise the possibility of tailoring an individualized therapeutic strategy based on the patients’ genetic and personal background (8).

Ashkenazi Jews (AJ) constitute a unique population to start implementing such genetic-based personalized approach since more than one-third of AJ-PD patients have a known mutation in either the *GBA* (eight mutations) or the *LRRK2* (G2019S) genes (9, 10). Herein, we will highlight some clinical features of *GBA* or *LRRK2*-associated PD among the AJ population and propose a new vision for personalized medicine based on genetic background. The immediate consequence of such genetic-based personalized approach is the need to genetically test for *GBA* and *LRRK2* mutations all recently diagnosed AJ-PD patients, patients entering clinical trials and those referred to deep brain stimulation (DBS). It is important to stress that the clinical genetic correlation we report here is based mostly on retrospective data and the proposed clinical approach has never been examined in prospective long-term care. We hope this paper will lead a scientific discussion and encourage prospective studies and will be the first step in the development of a personalized approach in the development of therapeutic strategies for patients with PD.

## *LRRK2*-G2019S PD

*LRRK2*-G2019S PD has been reported to manifest with a mild phenotype with less cognitive impairments (11, 12), higher frequencies of sleep onset insomnia, and equal frequencies of daytime sleepiness but less REM sleep behavior disorders (RBD) compared to idiopathic PD (iPD) (13, 14). *LRRK2*-G2019S PD patients seem to express better olfactory function compared to iPD patients (15) and appear to present more frequently with the postural instability gait difficulty (PIGD) sub-type (16), although in another publication tremor was identified as a distinguished feature of *LRRK2*-PD (17). Some discrepancies appear in the literature relating to the presence of higher prevalence of psychiatric symptoms in *LRRK2*-G2019S PD patients (18, 19) while other studies could not detect such a trend (11). Both higher and lower rates of depression in *LRRK2*-G2019S PD patients have been reported compared to iPD (17, 20).

## *GBA*-PD

Studies reported that heterozygous, homozygous, and compound-heterozygote *GBA*-PD patients present with more severe cognitive decline compared to iPD (21–23). The association between mutations in the *GBA* gene and early cognitive decline has recently received much support by observations that *GBA* carriers frequently develop Lewy Body Dementia (LBD), a syndrome that is tightly associated with PD and is one of the classical synucleopathies, defined by the development of clinically significant cognitive decline prior to, or at about the same time as, the appearance of parkinsonism (24). In addition, higher incidence of RBD, a known marker for cognitive decline in PD, was reported in *GBA*-PD patients (25) as well as more frequent psychiatric symptoms than in iPD (26). Carriers of severe *GBA* mutations were found to have an earlier onset PD symptoms (27) and more autonomic disturbances at PD diagnosis and as the disease progressed, reflecting earlier and more sever autonomic degeneration (27) (Table 1).

**Table 1:** **Differences in symptomatology based on genetic status as compared to iPD**.

	*LRRK2*	*GBA*
Age of onset	Similar to iPD (30)	Earlier than iPD (27)
Hyposmia	Better than iPD (15)	Significant (47, 53)
Cognitive decline	Better cognitive performance (11, 12)	Significant (22, 23, 54)
Depression	Reports of both higher and lower frequencies (17, 20)	More severe (55)
RBD	Less than iPD (13, 14)	More severe (53, 55)
Motor phenotype	PIGD (16, 29)	More postural instability (31)
Specific targeted pharmacological treatment	Ursedeoxycholic acid (56)	Ambroxol (57)
Autonomic	Less than iPD (58)	More severe involvement (23)
Dyskinesia	Same as iPD (39)	More severe compared to iPD (40)

### How PD Differs in Patients with *LRRK2* or *GBA* Mutations?

#### Early in the Clinical Course

##### Gait

Early postural instability and gait disturbances in PD is known to be associated with earlier falls, more freezing of gait (FOG) and more frequent cognitive decline (28). A large proportion of *LRRK2*-G2019S-PD patients were found to present with PIGD phenotype that is associated with higher risk of falls (29, 30). Similarly, *GBA-*PD mutation carriers demonstrated increased gait impairments and FOG, which has been associated with disturbed executive functions (31). Such decline in functional performance is an indirect marker of decreased motor-cognitive reserve, which should be taken into account while developing treatment strategies.

Parkinson disease patients, with either *LRRK2*-G2019S mutation or any mutation in the *GBA* gene, should be informed about their higher risk for earlier falls or FOG, and treatment should be offered to try and delay or prevent these serious motor complications. Such recommendations will include using extra caution when offering drugs that increase fall risk, such as anti-depressive drugs, benzodiazepines, hypnotics, anti-cholinergics and possibly dopamine agonists (DA) because of their effect on alertness (32, 33). In conjunction, physical or cognitive therapy should be recommended and encouraged as early as possible and reinforced in every visit. Prevention of falls and FOGs should be a priority in order to avoid the need for treatment of their destructive consequences.

### Early Cognition–Mood Changes

The tendency for earlier, faster, and more clinically meaningful cognitive decline in *GBA*-PD should be a major consideration in treatment strategy. In view of the negative impact of all anti-parkinsonian drugs on cognition, alertness, sleep, and the risk of psychosis (34), drug choices or drug combinations should be prescribed more cautiously than when treating iPD patients. In addition, cognitive state, sleep quality, mood (depression and anxiety), hallucinations, or delusions should be assessed in every visit of a *GBA*-PD patient, in an effort to establish early detection and to prevent sever psychosis, a leading cause for hospitalization and institutionalization (35). Intensive exercise programs and multi-domain cognitive enhancing interventions should be recommended early to enhance capacity and reserve, and the above knowledge should be used to increase adherence to both short-term and long-term behavioral modifications.

Such variability in disease course and in the risk of developing significant complications, as well as drug-induced side effects in *GBA*-PD, must be taken into consideration when including patients into any clinical trial, but especially in studies testing disease modifying drugs.

### Increased Risk of Experiencing Autonomic Disturbances

The higher rates of autonomic symptoms presented by *GBA*-PD patients (23) has major importance in treatment strategies since all anti-parkinsonian drugs can worsen or cause orthostatic hypotension while some can trigger urinary retention, delayed gastric emptying time, constipation, erectile dysfunction, delayed ejaculation, and lymphatic edema (36). The increased risk of early occurrence of clinically significant autonomic disturbances or treatment-induced autonomic complications should influence the therapeutic approach as well. Careful monitoring for early signs of autonomic dysfunctions in *GBA*-PD patients should be a recommended practice. Furthermore, the increased vulnerability of the autonomic nervous system should be taken into account when such patients are considered for participating in any drug or interventional clinical trials.

### Later Disease Stage Features

#### Dyskinesia

One of the most significant motor complication of dopaminergic treatment and especially levodopa is dyskinesia (37). Involuntary movements are frequently the biggest hurdle for effective dopaminergic treatment and the strategy to delay levodopa and use less effective substitutes, such as DA, was initiated specifically to delay these complications (38). *LRRK2*-PD patients were found to have equal frequencies of levodopa-induced dyskinesia compared to iPD (39). By contrast, it has been reported that *GBA*-PD patients are more sensitive to levodopa, developing dyskinesia earlier in the course of the disease (40). Combined with the earlier age of disease onset in *GBA-*PD (27), which is known to be associated with earlier and more aggressive Dopa-induced dyskinesia (41). *GBA-*PD patients should be considered from disease diagnosis to have increased risk for early disabling dyskinesia, lower dosages of levodopa and possibly, delaying levodopa should be common practice, even more than in the general PD population, in this group of *GBA*-PD patients.

### Should Mutation Status Influence Referrals to Deep Brain Stimulation?

The role of genotype in the outcome of DBS has not been well studied but it is possible that the risk to develop cognitive decline or gait disturbances, the two most common serious and long-term complications of DBS (42, 43) could be influenced by PD-associated mutations. Angeli et al. identified earlier cognitive decline in *GBA*-PD patients compared with controls (44), AJ *LRRK2*-PD patients had a similar outcome as iPD patients after subthalamic nucleus (STN) DBS (45), while a study in North African Arabs identified better outcome for *LRRK2*-PD patients after STN-DBS compared to iPD patients (46). Based on current knowledge, it could be speculated that more frequent behavioral and cognitive changes as well as earlier gait disturbances could be observed in PD patients, carriers of mutation in either the *GBA* or *LRRK2* genes after DBS.

To the best of our knowledge, the effect of DBS on gait was not explored in the context of patients’ genetic status. Because of its cardinal clinical consequences, it is important to explore retrospective cohorts and retrieve information about the short- and long-term clinical outcomes of DBS to the globus pallidus interna and STN in patients with iPD as well as those with known mutations. Until such data are available, referring PD patients with mutations in the *GBA* or the *LRRK2* genes to DBS should be done after special consideration and after sharing the potential risks with the patients and their families.

### Parkinsonism as the Manifesting Symptom of GD

Recent investigation into the association of Gaucher disease (GD) and PD has indicated that parkinsonism could be the first neurological symptom of GD (47). In addition, our group has recently assessed 1100 AJ-PD patients and found 12 subjects who were either homozygous or compound-heterozygous for mutations in the *GBA* gene, half of which were not aware of any GD symptoms (ahead of print). This has important implications regarding the potential for reduced bone density and increased fractures following falls, which is a further reason for genetic screening of the AJ population.

### Therapeutic Approach based on Genetic Testing

Throughout the course of PD, many pharmacological options as well as non-pharmacological interventions are available for the treatment of disease symptoms. Decisions should be made regarding the first anti-parkinsonian drug to prescribe, whether to add DA or levodopa or rather start treatment with levodopa right away, how to treat depression, anxiety, and sleep disturbances and how to monitor autonomic dysfunctions. Later on, questions regarding the combination of drugs, the use of anti-cholinergic drugs, or cholinesterase inhibitors for treating cognitive decline or sleep impairments and RBD, and the use of atypical dopamine-blocking agents, such as quetiapine or clozapine, should be addressed. In addition, the intensity and quality of physiotherapy, cognitive enhancement therapies, and falls prevention strategies are all available but, currently, not uniformly recommended and implemented. Lastly, a recent epidemiological study exploring environmental and behavioral habits, such as caffeine consumption, found a possible risk-reducing interaction between caffeine consumption and *LRRK2-*PD (48). Yet, patients’ engagement in life-style modification strategies is highly variable, highly associated with the level of knowledge and commitment of patients and care-givers to the recommended non-pharmacological interventions.

We believe that the present knowledge about the different course of PD among AJ and non-Jewish patients based on *GBA* and *LRRK2* mutations’ status and the extremely high frequency of mutation carriers in the AJ-PD patients (35%) justifies early genetic screening and tailoring treatment approach based on patients’ genotypes. However, the reduced penetrance rates for these mutations (49, 50) suggest that other modifier genes may be responsible for both increased and reduced risk of developing PD in this unique population (51, 52) as well as other aspects of the disease and demand further elucidation. We further recommend that the genotype needs to be considered when recruiting patients into clinical trials especially when examining the efficacy of novel drugs. Such approach could foster the recruitment of a smaller sample size in fewer centers. Moreover, exploring efficacy of a drug with specific biological target, such as on lysosomal activity should likely be first tested in patients with mutations in the *GBA* gene.

In the present paper, we lumped together our discussion on *GBA* mutation carriers although we believe that specific mutations may influence disease course and also the effect of medications. Currently, there is only limited information to support splitting the recommendations according to individual mutations, or as a group of “mild” or “severe” mutations. However, future studies will likely lead to refinement of this approach also within *GBA*-PD.

In conclusion, we believe that the available evidence in regard to the specific course of *GBA* or *LRRK2* mutations’ associated PD is sufficient to recommend genotyping new PD patient (especially those with AJ background). Such genotyping should influence the therapeutic approach throughout the course of the disease and should also be considered when recruiting patients into clinical trials.

## Author Contributions

NG – conceptualization and writing, AM – writing, AT – writing, AO-U – writing. All authors agree to be accountable for the content of this work. All co-authors have read and approved the content of this manuscript. This manuscript is not under review of any other journal.

## Conflict of Interest Statement

NG serves as a member of the Editorial Board for the Journal of Parkinson’s disease. He serves as consultant to Teva-Lundbeck, IntecPharma, NeuroDerm, Armon Neuromedical Ltd\Dexel, Monfort and Lysosomal Therapeutic Inc. He received payment for lectures at Teva-Lundbeck, Novartis, UCB, Abviee, Shaier, and Genzyme. Prof. NG received research support from the Michael J Fox Foundation, the National Parkinson Foundation, and the Israel Science Foundation. AM – nothing to disclose. AT – nothing to disclose. AO-U – nothing to disclose.
